# Energy-Efficient 3D Trajectory Optimization and Resource Allocation for UAV-Enabled ISAC Systems

**DOI:** 10.3390/e28020248

**Published:** 2026-02-21

**Authors:** Lulu Jing, Hai Wang, Zhen Qin, Yicheng Zhao, Yi Zhu, Wensheng Zhao

**Affiliations:** 1 College of Communications Engineering, Army Engineering University, Nanjing 210042, China; jinglulu@njfu.edu.cn (L.J.);; 2 Office of Cyber Security and Informatization, Nanjing Forestry University, Nanjing 210037, China; 3 Department of Information and Communication, Noncommissioned Officer Academy of PAP, Hangzhou 310000, China; 4 Academy of Systems Engineering, Academy of Military Sciences, Beijing 100091, China

**Keywords:** UAV, ISAC system, energy efficiency, 3D trajectory, resource allocation

## Abstract

Owing to their high flexibility, autonomous operation, and rapid deployment capability, unmanned aerial vehicles (UAVs) serve as effective aerial platforms for sensing and communication in remote and time-critical scenarios. However, their limited onboard energy budget poses a significant bottleneck for sustained operations. This paper investigates an energy-efficient UAV-assisted integrated sensing and communication (ISAC) system, aiming to maximize the sensing energy efficiency (SEE), defined as the ratio of the total radar estimation rate to the total energy consumption. Unlike prior works focused solely on rate maximization or fairness, our design jointly optimizes the UAV’s 3D trajectory, task scheduling, and power allocation under kinematic and coverage constraints to maximize the SEE. To solve the formulated non-convex fractional programming problem, we propose an efficient iterative algorithm based on the Dinkelbach method and block coordinate descent (BCD). Simulation results demonstrate that the proposed scheme achieves a superior trade-off between sensing performance and energy consumption.

## 1. Introduction

### 1.1. Background

Unmanned aerial vehicles have found widespread application in various fields due to their mobility, flexibility, and rapid deployment [1,2]. In many real-time control systems, however, the timely and reliable transmission of control commands is essential, requiring strong sensing capabilities and extremely low communication latency [3]. These requirements become even more critical in emergency communication scenarios, where ground IoT devices or conventional infrastructure may be damaged or unavailable due to disasters or unexpected events. Although deploying separate communication and sensing payloads on UAVs can enhance functionality, this approach increases hardware burden and spectrum consumption [4]. To overcome these challenges, integrated sensing and communication (ISAC) technology has been developed, allowing UAVs to perform wireless information transfer and environmental sensing simultaneously using shared resources [5,6]. Recent advances in UAV-enabled ISAC have demonstrated significant improvements in both communication and sensing performance, particularly in resource-constrained and mission-critical environments.

The development of UAV-assisted ISAC technology aims to reduce device redundancy and enhance spectrum utilization, making it a key research direction for the future of IoT [7,8,9]. In the context of ISAC services for the IoT, the UAV typically acts as a dual-function aerial base station, both sensing environmental information and transmitting this data to ground IoT devices. As ISAC technology advances, radar and communication signals are increasingly integrated, enabling shared spectrum and reducing equipment redundancy. This integration not only greatly improves spectrum efficiency but also provides flexible and efficient sensing and communication capabilities for a wide range of IoT applications.

### 1.2. Related Work

Motivated by the growing importance of UAV-enabled ISAC in IoT applications, numerous studies have explored its key challenges and potential solutions. In [10], the authors proposed a UAV-assisted ISAC strategy, where the extended Kalman filter was employed to fuse communication-based location and sensing information, thereby enhancing target sensing accuracy, while an ISAC-based Identification Friend or Foe (IFF) method was developed to reduce communication delay. In [11], a network utility maximization problem was studied in a dual-functional radar-communication multi-UAV network, where UAVs jointly served communication users and cooperatively performed target sensing. The authors formulated a joint optimization problem involving UAV positioning, user association, and transmission power control to balance communication and sensing performance. In [12], an integrated scheduling framework for sensing, communication, and motion control was proposed for mmWave/THz UAV networks to support high-rate backhaul data transmission. The authors analyzed the strong coupling between sensing and motion control, introducing a novel state-to-noise-ratio metric that links the activation of sensing-control patterns with beam alignment data rates and derived a closed-form expression for the joint design, ensuring both mmWave/THz data rate requirements and UAV motion control performance. In [13], a UAV relaying network with ISAC capabilities was proposed to address spectrum congestion and blockage scenarios. The authors introduced a sensing-weighted algorithm that jointly designs BS transmit beamforming and UAV amplification factors to enhance sensing accuracy, maximize achievable rate, and minimize system outage.

The above studies mainly focus on ISAC for static UAVs and overlook the mobility characteristics of UAVs. To further exploit the advantages of UAV mobility, the following works have investigated ISAC applications in scenarios involving mobile UAVs. In [14], Lin et al. proposed a UAV-assisted ISAC system to address the conflicting requirements of high-quality communication and low-latency sensing in 6G networks. They introduced a flight-hover-communication protocol, where the UAV communicates with IoT devices while hovering and performs sensing during flight, and developed a deep reinforcement learning-based trajectory planning algorithm to maximize device connectivity and minimize UAV energy consumption. In [15], Liu et al. proposed a multi-UAV-enabled ISAC system for IoT networks, introducing radar mutual information as a metric for sensing performance. They formulated a joint optimization problem to maximize the minimum communication rate per IoT node by optimizing node scheduling, transmit power, and UAV 3D trajectory under radar MI constraints. The problem was decomposed into three subproblems, and a three-layer iterative algorithm was developed to obtain near-optimal solutions. In [16], Meng et al. proposed an integrated periodic sensing and communication (IPSAC) mechanism for UAV-enabled ISAC systems to address the challenge of asymmetric sensing and communication requirements. They aimed to maximize the system achievable rate by jointly optimizing UAV trajectory, user association, target sensing selection, and transmit beamforming, subject to sensing frequency and beam pattern gain constraints. In [17], Lyu et al. proposed a UAV-enabled ISAC system in which UAVs serve as aerial dual-functional access points for simultaneous communication and sensing. They considered both quasi-stationary and fully mobile UAV scenarios, and formulated the joint optimization of UAV deployment or trajectory and transmit beamforming to maximize the weighted sum-rate of users.

Beyond throughput, maintaining the timeliness of sensing data is another critical performance metric in UAV-assisted ISAC systems [2,18]. To evaluate the freshness of sensing data, the AoI (Age of information, AoI) has been widely adopted as a key performance metric, and several studies have investigated AoI in UAV-assisted ISAC systems. In [19], Zhu et al. studied AoI-optimal trajectory planning for UAVs in ISAC networks. They used the UAV’s geometric position as an anchor in the trajectory design and applied dynamic programming to optimize the communication AoI while maintaining positioning performance. In [20], He et al. investigated a UAV-assisted system integrating sensing and communication, with the objective of minimizing the AoI of sensing data. The authors jointly optimized UAV scheduling, transmit beamforming, trajectory, and power under communication quality and physical constraints, and derived a closed-form solution for the beamforming design. In [21], Mei et al. studied an air-ground integrated sensing and communication network with UAVs, focusing on minimizing the AoI under jamming attacks. They jointly optimized the trajectory and transmit power of the mission UAV to mitigate the impact of mobile UAV attackers and formulated the problem as a dynamic, NP-hard optimization. They then proposed a TD3-based deep reinforcement learning algorithm for trajectory and power control, incorporating reward shaping to accelerate convergence. In [22], a UAV-assisted ISCC system was proposed, where the UAV performs sensing, computation, and communication for remote users. The authors introduced AoI and radar estimation rate as key metrics to evaluate information freshness and sensing quantity. However, minimizing AoI usually requires frequent updates and intensive maneuvers, which inflate propulsion energy consumption. Pursuing very low AoI or very high throughput in isolation often yields poor efficiency. Therefore, it is necessary to adopt a metric that balances sensing performance against efficiency expenditure.

Motivated by this tradeoff, substantial research has explored resource allocation within ISAC systems. Notably, reference [23] proposes a value-of-service (VoS)-oriented scheme to balance efficiency and fairness in multi-user ISAC through a bargaining game approach. Similarly, reference [24] tackles throughput maximization in fairness-aware cooperative sensing systems by jointly optimizing beamforming and time allocation. However, these studies predominantly target conventional communication metrics like throughput or fairness. A critical limitation in these approaches, particularly for UAV-enabled scenarios, is the oversight of propulsion energy consumption, which typically dominates the total energy budget. By neglecting the substantial energy required to sustain flight, existing frameworks fail to provide a comprehensive energy efficiency assessment for mobile UAV platforms. This creates a pressing need for a holistic design objective that explicitly accounts for the intricate coupling between 3D propulsion dynamics and ISAC performance.

In this paper, we study the energy-efficient design for a UAV-assisted ISAC system, where the UAV acts as an aerial platform providing sensing and communication services to ground targets. This architecture is particularly advantageous when conventional ground infrastructure is damaged or unavailable, such as in post-disaster scenarios or remote areas. In such cases, the UAV serves as a flexible platform to enable real-time sensing and reliable data upload to the collection center. However, the performance of such systems is fundamentally constrained by the UAV’s finite onboard energy. A critical design challenge lies in the trade-off between sensing performance and energy consumption. Intuitively, to maximize the radar estimation rate, the UAV should fly at a low speed or hover near the targets to maintain favorable channel conditions. However, such maneuvers are often inefficient in terms of propulsion energy consumption, especially for fixed-wing UAVs. Therefore, the energy-efficient trajectory design needs to strike an optimal balance between maximizing the sensing yield and minimizing the UAV’s energy consumption. To this end, the proposed model aims to maximize the sensing energy efficiency, achieved by jointly optimizing the UAV’s 3D trajectory, task scheduling, and power allocation under strict energy budget constraints. The contributions of this paper are summarized as follows.

- A UAV-assisted integrated sensing and communication (ISAC) framework is proposed, with the novel introduction of sensing energy efficiency (SEE) as its core performance metric. Unlike prior works that treat throughput and energy separately, SEE holistically quantifies the mission sustainability by defining the system-wide trade-off as the ratio of the total radar estimation rate to the total energy consumption, which incorporates both communication and 3D propulsion costs.
- To achieve energy-efficient sensing under the stringent onboard battery capacity, we formulate a joint optimization problem that directly maximizes SEE rather than merely maximizing rate or minimizing energy. Unlike traditional throughput-oriented designs, this formulation explicitly balances the trade-off between maximizing the sensing data and minimizing the propulsion energy cost. The problem jointly optimizes the UAV’s 3D trajectory, task scheduling, and power allocation under unified kinematic and coverage constraints, enabling a truly integrated design.
- To solve the resulting non-convex fractional problem efficiently, we develop a dedicated two-layer algorithmic framework. The outer layer employs the Dinkelbach method. Crucially, the inner layer utilizes the Quadratic Transform (QT) method to decouple the complex fractional terms in the objective, followed by a Block Coordinate Descent (BCD) framework where each subproblem—scheduling, power allocation, and trajectory planning—is solved via Successive Convex Approximation (SCA), ensuring convergent and computationally efficient coordination.

The remainder of this paper is organized as follows. Section 2 establishes the energy-constrained UAV-assisted ISAC framework and details the data transmission and radar detection processes. It explicitly derives the UAV propulsion and transmission energy consumption models, defines the sensing energy efficiency metric, and formulates a joint optimization problem to maximize energy efficiency under kinematic and coverage constraints. Section 3 presents a comprehensive two-layer iterative optimization algorithm, combining the Dinkelbach method with Block Coordinate Descent and Successive Convex Approximation to efficiently solve the formulated non-convex fractional programming problem. Section 4 provides numerical results to validate the effectiveness of the proposed sensing energy efficiency design, and Section 5 concludes the paper.

## 2. System Model and Problem Formulation

### 2.1. Scenario Description and UAV Kinematics

**Figure 1:**
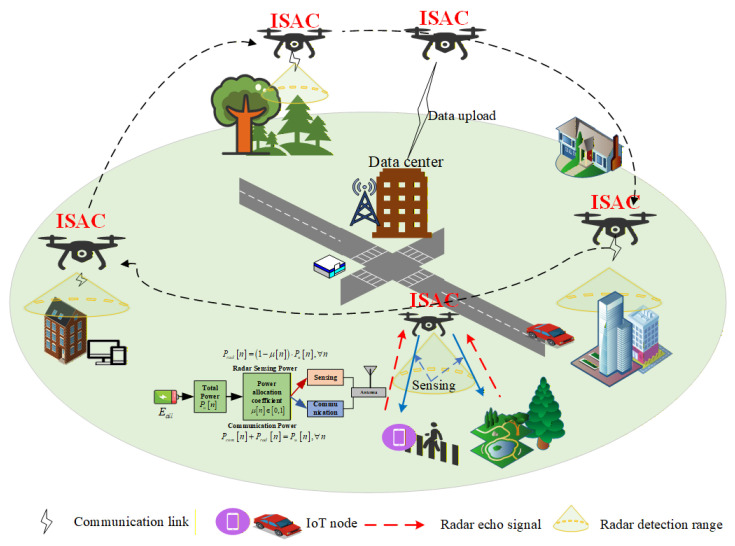
Example of an ISAC system.

As shown in Figure 1, we consider a UAV-assisted IoT detection system based on an integrated sensing and communication framework. The system consists of a single UAV, a data collection center, and K IoT nodes, denoted by where $k\in K=\left\{1,2,\cdots ,K\right\}$. These nodes are randomly distributed around the data collection center. In this framework, the UAV acts as a mobile ISAC platform responsible for environmental monitoring and data relaying. Specifically, when the UAV is within the radar detection range of a target node, it performs radar sensing to capture environmental data surrounding that node and simultaneously transmits the acquired information to it. Conversely, when the target nodes are outside the radar’s field of view, the UAV executes a data upload task, transmitting the stored sensing data to the data collection center via the communication uplink. Let the fixed ground locations of the data collection center and the IoT node be $l_{c}=\left[g_{c},H_{c}\right]$, $l_{k}=\left[g_{k},H_{k}\right]$, where $g_{c}=\left(x_{c},y_{c}\right)$, $g_{k}=\left(x_{k},y_{k}\right)$, denote the horizontal coordinates, and $H_{c}$, $H_{k}$, represent the altitudes of data collection center and IoT nodes, respectively.

Assuming a UAV performs ISAC and data transmission tasks over a flight period T, the flight period T is discretized into N slots of length, and slot index δ=T/N. Depending on target detection, the UAV switches between ISAC and data upload modes. Consequently, the UAV’s trajectory can be denoted as $l_{u}=\left[q_{u}\left[n\right],H_{u}\left[n\right]\right]$, where $q\left[n\right]=\left(x_{u}\left[n\right],y_{u}\left[n\right]\right)$ and $H_{u}\left[n\right]$ represent horizontal position and flight altitude, respectively.

For operational feasibility, the duration is selected to satisfy the minimum time requirements for tasks, while being sufficiently small to approximate the trajectory within each slot as a linear segment. Let $v\left[n\right]=\left[V_{xy}\left[n\right],V_{z}\left[n\right]\right]$ and $a\left[n\right]=\left[a_{xy}\left[n\right],a_{z}\left[n\right]\right]$ denote the velocity and acceleration vectors, respectively. Furthermore, the trajectory is subject to kinematic constraints and a cyclic boundary condition requiring identical initial and final states. These constraints are formulated as follows:

(1) $$q\left[n+1\right]=q\left[n\right]+V_{xy}\left[n\right]\delta ,\forall n$$

(2) $$q\left[0\right]=q_{0},q\left[N+1\right]=q_{F}$$

(3) $$V_{xy}\left[n+1\right]=V_{xy}\left[n\right]+a_{xy}\left[n\right]\delta ,\forall n$$

(4) $$V_{z}\left[n+1\right]=V_{z}\left[n\right]+a_{z}\left[n\right]\delta ,\forall n$$

(5) $$v\left[0\right]=v_{0},v\left[N+1\right]=v_{F}$$

(6) $$V_{xy}^{\min}\leq V_{xy}\left[n\right]\leq V_{xy}^{\max},\forall n$$

(7) $$V_{z}^{\min}\leq V_{z}\left[n\right]\leq V_{z}^{\max},\forall n$$

(8) $$a_{xy}\left[n\right]\leq a_{xy}^{\max},a_{z}\left[n\right]\leq a_{z}^{\max},\forall n$$

(9) $$H_{u}\left[n+1\right]=H_{u}\left[n\right]+V_{z}\left[n\right]\delta$$

(10) $$H\left[1\right]=H\left[N\right]$$

(11) $$H_{\min}\leq H_{u}\left[n\right]\leq H_{\max}$$

where $V_{xy}^{\max},V_{xy}^{\min}$, $a_{xy}^{\max},a_{z}^{\min}$ and $H_{\min},H_{\max}$ denote the maximum or minimum of velocity, acceleration and altitude of the UAV, respectively, $\left\|•\right\|$ represents the $L_{2}$ norm, and (2), (5), (10) enforces that the UAV’s initial and terminal status are identical. Equations (1), (3), (4) and (9) represent the discrete-time state evolution of the UAV based on Newtonian kinematics. These equality constraints ensure the continuity of the UAV’s position and velocity vectors across consecutive time slots, encompassing both the horizontal plane $q\left[n\right]=\left(x_{u}\left[n\right],y_{u}\left[n\right]\right)$ and the vertical dimension $H_{u}\left[n\right]$. Equations (2), (5) and (10) enforce the cyclic boundary conditions. These constraints require the UAV to return to its initial state (position, velocity, and altitude) at the end of the flight period T. Inequalities (6)–(8) and (11) define the feasible kinematic region constrained by the UAV’s mechanical performance and flight safety requirements.

### 2.2. UAV Task Scheduling Model

To characterize the discrete decision-making process of the UAV, we introduce two sets of binary scheduling variables. Let $\omega_{k}\left[n\right]$ denote the ISAC task indicator for the node at time slot n, and let denote the data upload task indicator. Specifically, $\omega_{k}\left[n\right]=1$ implies that the UAV executes the ISAC task for node k, while $\alpha \left[n\right]=1$ indicates that the UAV uploads the sensing data to the data collection center. To avoid task overlap, the two variables satisfy

(12) $$\alpha \left[n\right]\in \left\{0,1\right\},\forall k,n$$

(13) $$\omega_{k}\left[n\right]\in \left\{0,1\right\},\forall k,n$$

(14) $$\sum_{k=1}^{K}\omega_{k}\left[n\right]+\alpha \left[n\right]=1,\forall n$$

Equation (14) imposes a strictly exclusive scheduling constraint; at any slot, the UAV must dedicate its resources solely to one specific task, either performing radar sensing on a target node or uploading data to the collection center.

Furthermore, the execution of the ISAC task is spatially constrained by the radar’s field of view. The UAV can only schedule an ISAC task for a node if the node falls within the radar’s coverage cone, defined by a maximum detection angle θ. Consequently, the scheduling variable $\omega_{k}\left[n\right]$ is coupled with the UAV’s 3D position by the following geometric constraint:

(15) $$\omega_{k}\left[n\right]{\left\|q\left[n\right]-g_{k}\right\|}^{2}\leq {\left(H_{u}\left[n\right]\tan\theta \right)}^{2},\forall k,n$$

It mandates that the UAV’s horizontal distance to the node must be within the instantaneous coverage radius $H_{u}\left[n\right]\tan\theta$ only when $\omega_{k}\left[n\right]=1$.

### 2.3. UAV Power Allocation Model

For a UAV equipped with ISAC capability, the transmit power in each time slot is shared between communication and radar sensing. Let $P_{u}\left[n\right]$ denote the total transmit power of the UAV in the slot n, and let $P_{rad}\left[n\right]$, $P_{com}\left[n\right]$ denote the portions assigned to data communication and radar sensing, respectively. We adopt a power allocation model such that

(16) $$P_{com}\left[n\right]+P_{rad}\left[n\right]=P_{u}\left[n\right],\forall n$$

Define $\mu \left[n\right]\in \left[0,1\right]$ as the power allocation coefficient in the time slot n. Then the communication and sensing power are given by

(17) $$P_{com}\left[n\right]=\mu \left[n\right]\cdot P_{u}\left[n\right],\forall n$$

(18) $$P_{rad}\left[n\right]=\left(1-\mu \left[n\right]\right)\cdot P_{u}\left[n\right],\forall n$$

The coefficient $\mu \left[n\right]$ is active only in ISAC slots, where the UAV simultaneously supports sensing and communication for ground nodes. In data upload slots, sensing is disabled and we set $\mu \left[n\right]=1$, so that all transmit power is assigned to the uplink communication with the data–collection center.

### 2.4. UAV Integrated Sensing and Communication Model

We consider two types of air-to-ground channels: ISAC channels between the UAV and each IoT node, which are used for both communication and radar sensing; a data-upload channel between the UAV and the data collection center. All these links are assumed to be dominated by line-of-sight (LoS) propagation. Empirical studies (e.g., [25]) show that when the flight altitude is around 100 m or higher, the probability of LoS connectivity for UAV–ground channels approach 100%. Let $d_{c}^{2}\left[n\right]={\left\|q\left[n\right]-g_{c}\right\|}^{2}+{\left(H_{u}\left[n\right]-H_{c}\right)}^{2}$ and $d_{k}^{2}\left[n\right]={\left\|q\left[n\right]-g_{k}\right\|}^{2}+{\left(H_{u}\left[n\right]-H_{k}\right)}^{2}$ denote the squared distances from the UAV to the data collection center and node k, respectively.

(19) $$h_{center}\left[n\right]=\frac{G_{t}G_{c}\lambda^{2}}{{\left(4\pi \right)}^{2}d_{c}^{2}\left[n\right]}=\frac{\beta_{0}}{d_{c}^{2}\left[n\right]},\forall k,n$$

where $G_{t}$ and $G_{c}$ denote the antenna gains of the UAV transmitter and communication receiver, respectively; $\lambda =c/f_{0}$ denotes the signal wavelength, where c and $f_{0}$ denote the speed of light and signal carrier frequency, respectively; $\beta_{0}=G_{t}G_{c}\lambda^{2}/{\left(4\pi \right)}^{2}$ represents the reference channel gain at position 1 m [5].

(20) $$h_{k,com}\left[n\right]=\frac{G_{t}G_{c}\lambda^{2}}{{\left(4\pi \right)}^{2}d_{k}^{2}\left[n\right]}=\frac{\beta_{0}}{d_{k}^{2}\left[n\right]},\forall k,n$$

(21) $$h_{k,rad}\left[n\right]=\frac{G_{t}G_{r}\lambda^{2}\sigma }{{\left(4\pi \right)}^{3}d_{k}^{4}\left[n\right]}=\frac{\beta_{r}}{d_{k}^{4}\left[n\right]},\forall k,n$$

where $G_{r}$ denotes the antenna gains of the UAV radar receiver [26], σ represents the radar cross-section (RCS) of the target nodes, and $\beta_{r}=G_{t}G_{r}\lambda^{2}\sigma /{\left(4\pi \right)}^{3}$.

To provide a unified and comparable basis for evaluating both communication and sensing performance in the UAV-assisted ISAC system, we adopt two information-theoretic metrics. The information rate quantifies the communication efficiency between the UAV and its targets (sensor nodes or data center), whereas the radar estimation rate reflects the UAV’s sensing capability by measuring the amount of target-related information encoded in the radar echo. A higher radar estimation rate indicates a greater amount of sensing information embedded in the radar echo signals. As mentioned earlier, the UAV’s transmission power is divided between communication and radar sensing tasks within the same time slot, which inherently causes self-interference between the two functions. To accurately reflect this coupling effect, the SINRs (Signal-to-Interference-plus-Noise ratio, SINR) for both communication and radar sensing links are defined as

(22) $$\Gamma_{k,com}\left[n\right]=\frac{P_{com}\left[n\right]h_{k,com}\left[n\right]}{P_{rad}\left[n\right]h_{k,com}\left[n\right]+N_{0}B},\forall k,n$$

(23) $$\Gamma_{k,rad}\left[n\right]=\frac{P_{rad}\left[n\right]h_{k,rad}\left[n\right]}{P_{com}\left[n\right]h_{k,rad}\left[n\right]+N_{0}B},\forall k,n$$

where $N_{0}$ is the power spectral density and B is the bandwidth of the signal. Based on [22,27], the information rate of the communication link between the UAV and its target at the time slot n can be mathematically expressed as

(24) $$R_{center}\left[n\right]=\log_{2}\left(1+\frac{P_{u}\left[n\right]h_{center}\left[n\right]}{N_{0}B}\right),\forall n$$

(25) $$R_{k,com}\left[n\right]=\log_{2}\left(1+\Gamma_{k,com}\left[n\right]\right),\forall k,n$$

(26) $$R_{k,rad}\left[n\right]=\log_{2}\left(1+\Gamma_{k,rad}\left[n\right]\right),\forall k,n$$

To ensure reliable information delivery without data loss, the system must satisfy both real-time and aggregate throughput requirements. Specifically, the amount of communication information transmitted to the nodes in each time slot should be no less than that of radar sensing information. Moreover, over the entire flight cycle, the amount of communication information transmitted to the data collection center should be no less than the total amount of radar sensing information for all the nodes. Hence, the communication constraints can be denoted as

(27) $$R_{k,rad}\left[n\right]\leq R_{k,com}\left[n\right],\forall k,n$$

(28) $$\sum_{n=1}^{N}\sum_{k=1}^{K}\omega_{k}\left[n\right]R_{k,rad}\left[n\right]\leq \sum_{n=1}^{N}\alpha \left[n\right]R_{center}\left[n\right],\forall k,n$$

### 2.5. UAV Energy Consumption Model

To use the limited onboard energy of the UAV more efficiently, we explicitly model its energy consumption. For a fixed-wing UAV equipped with ISAC, the total energy expenditure consists of task energy (for ISAC or data-upload operations) and propulsion energy (for sustaining flight). Following the model in [28], the cumulative task and propulsion energies over all time slots can be expressed as

(29) $$E_{t}=\sum_{n=1}^{N}\delta P_{u}\left[n\right]$$

(30) $$E_{f}=\sum_{n=1}^{N}\delta \left(c_{1}{\left\|v\left[n\right]\right\|}^{3}+\frac{c_{2}}{\left\|v\left[n\right]\right\|}\left(1+\frac{{\left\|a\left[n\right]\right\|}^{2}}{g^{2}}\right)\right)$$

where $c_{1}$ and $c_{2}$ capture effects of the UAV hardware and flight environment, and g denotes gravitational acceleration, treated as a constant. Equation (30) models the propulsion power consumption for a fixed-wing UAV based on aerodynamic principles. The total energy consumption of the UAV is

(31) $$E_{t}+E_{f}=E_{all}$$

where $E_{t}$ and $E_{f}$ denote the task and propulsion energies in slot n, respectively.

### 2.6. Problem Integration

In the considered energy-constrained UAV-assisted ISAC framework, the UAV periodically flies over a set of designated ground nodes to perform ISAC tasks and upload the collected sensing data to the data collection center. To use the limited onboard energy more efficiently, we formulate an energy-efficient joint optimization problem that integrates the UAV trajectory $L=\left\{q\left[n\right],H_{u}\left[n\right],v\left[n\right],a\left[n\right],\forall n\right\}$, the sensing and data upload scheduling strategy $S=\left\{\omega_{k}\left[n\right],\alpha \left[n\right],\forall n,k\right\}$, and the power allocation strategy $P=\left\{P_{u}\left[n\right],\mu \left[n\right],\forall n\right\}$. We adopt Sensing Energy Efficiency (SEE) as the performance metric. SEE is defined as the ratio of the total valid radar estimation rate to the total energy consumption:

(32) $$\eta_{SEE}=\frac{\sum_{n=1}^{N}\sum_{k=1}^{K}\omega_{k}\left[n\right]R_{k,rad}\left[n\right]}{E_{t}+E_{f}}$$

Our objective is to maximize $\eta_{SEE}$ by jointly optimizing the UAV’s task scheduling S, power allocation P and 3D trajectory L. The optimization problem is formulated as follows:

(33) $$P1:\underset{L,S,P}{\max\;}\eta_{SEE}$$

(33a) $$s.t.\;q\left[n+1\right]=q\left[n\right]+V_{xy}\left[n\right]\delta ,\forall n$$

(33b) $$q\left[0\right]=q_{0},q\left[N+1\right]=q_{F}$$

(33c) $$V_{xy}\left[n+1\right]=V_{xy}\left[n\right]+a_{xy}\left[n\right]\delta ,\forall n$$

(33d) $$V_{z}\left[n+1\right]=V_{z}\left[n\right]+a_{z}\left[n\right]\delta ,\forall n$$

(33e) $$v\left[0\right]=v_{0},v\left[N+1\right]=v_{F}$$

(33f) $$V_{xy}^{\min}\leq V_{xy}\left[n\right]\leq V_{xy}^{\max},\forall n$$

(33g) $$V_{z}^{\min}\leq V_{z}\left[n\right]\leq V_{z}^{\max},\forall n$$

(33h) $$a_{xy}\left[n\right]\leq a_{xy}^{\max},a_{z}\left[n\right]\leq a_{z}^{\max},\forall n$$

(33i) $$H_{u}\left[n+1\right]=H_{u}\left[n\right]+V_{z}\left[n\right]\delta$$

(33j) $$H\left[1\right]=H\left[N\right]$$

(33k) $$H_{\min}\leq H_{u}\left[n\right]\leq H_{\max},\forall n$$

(33l) $$\alpha \left[n\right]\in \left\{0,1\right\},\forall k,n$$

(33m) $$\omega_{k}\left[n\right]\in \left\{0,1\right\},\forall k,n$$

(33n) $$\omega_{k}\left[n\right]{\left\|q\left[n\right]-g_{k}\right\|}^{2}\leq {\left(H_{u}\left[n\right]\tan\theta \right)}^{2},\forall k,n$$

(33o) $$\mu \left[n\right]\in \left[0,1\right],\forall n$$

(33p) $$\;0\leq \frac{1}{N}\sum_{n=1}^{N}P_{u}\left[n\right]\leq P_{avg},\forall n$$

(33q) $$R_{k,rad}\left[n\right]\leq R_{k,com}\left[n\right],\forall k,n$$

(33r) $$\sum_{n=1}^{N}\sum_{k=1}^{K}\omega_{k}\left[n\right]R_{k,rad}\left[n\right]\leq \sum_{n=1}^{N}\alpha \left[n\right]R_{center}\left[n\right],\forall k,n$$

Problem P1 is a nonconvex fractional programming problem involving integer variables and coupled constraints. Obtaining a direct solution is intractable; therefore, we propose an efficient iterative algorithm in the subsequent section.

## 3. Proposed Solution Algorithm

The optimization problem P1 is a mixed-integer non-convex fractional program (MINFP). The intractability arises from three primary sources: the objective function is a ratio of two functions, which is non-linear and non-concave; the optimization variables for S, P, L are tightly coupled in the objective and constraints; both the objective and several constraints are nonconvex due to propulsion energy and rate functions.

To address this, we propose an efficient two-layer iterative algorithm. The outer layer employs Dinkelbach algorithm to transform the fractional objective into an equivalent subtractive form [29]. The inner layer utilizes a Block Coordinate Descent framework to solve the resulting non-convex problem, which is further decomposed into three manageable subproblems, each solved using Successive Convex Approximation techniques.

### 3.1. Outer Layer Dinkelbach Algorithm for P1

Problem (P1) is a fractional program that can be solved by iteratively solving a series of equivalent parametric problems. To tackle the non-convexity of the fractional objective function in (P1), we employ a Quadratic Transform method to decouple the numerator and denominator. This approach introduces an auxiliary variable ρ to reformulate the original problem into an equivalent parametric subtractive form. Let $A=\sum_{n=1}^{N}\sum_{k=1}^{K}\omega_{k}\left[n\right]R_{k,rad}\left[n\right]$, $B=E_{t}+E_{f}$, ρ is a parameter. The equivalent objective function $F\left(S,P,L,\rho \right)$ is constructed as shown in Equation (34):

(34) $$F\left(S,P,L,\rho \right)=2\rho \sqrt{A}-\rho^{2}B,\;\rho =\frac{\sqrt{A}}{B}$$

The proof of the equivalence between maximizing this auxiliary function $F\left(S,P,L,\rho \right)$ and the original fractional objective is provided in Appendix B.

This allows us to replace the fractional problem (P1) with the iterative optimization of the subtractive problem (P2). In each outer iteration i, given the value $\rho^{\left(j\right)}$, we solve

(35) $$P2:\underset{L,S,P,\rho }{\max}2\rho^{\left(j\right)}\sqrt{A}-\rho^{2\left(j\right)}B$$

(35a) $$s.t.\;\left(33a\right)-\left(33r\right)$$

The outer loop continues until the objective problem (P2) converges, when the change in the objective value between two consecutive iterations is below a prescribed tolerance γ.

### 3.2. Inner Layer: Block Coordinate Descent for Problem P2

Although the Dinkelbach transformation removes the fractional structure, the problem P2 remains a challenging nonconvex optimization problem due to nonconvex constraints. To efficiently solve the problem P2, we employ a BCD framework that decomposes the joint optimization into three sequential subproblems.

#### 3.2.1. UAV Task Scheduling Optimization

Given the current power allocation P, L, we optimize the scheduling variables S to maximize the sensing performance. Under fixed power allocation P and trajectory parameters L, the term A is linear, the energy term B are fixed.

The scheduling subproblem P2.1 can be formulated as:

(36) $$P2.1:\underset{S}{\max}2\rho^{\left(j\right)}\sqrt{A}-\rho^{2\left(j\right)}B$$

(36a) $$s.t.\left(33l\right)-\left(33o\right),\left(33r\right)$$

which is a Mixed-Integer Linear Programming (MILP) problem that can be solved by Gurobi 12.0 directly.

#### 3.2.2. UAV Power Allocation Optimization

Given the updated scheduling S and the current trajectory L, we now optimize the power allocation variables P to maximize the objective function while satisfying power and rate constraints. With fixed S and L, the propulsion energy is constant and can be ignored. The subproblem P2.2 can be written as:

(37) $$P2.2:\underset{P}{\max}2\rho^{\left(j\right)}\sqrt{A}-\rho^{2\left(j\right)}B$$

(37a) $$s.t.\;\left(33p\right)-\left(33r\right)$$

This subproblem addresses the critical challenge of optimal resource allocation within the UAV’s power-constrained architecture. The optimization of $P_{u}\left[n\right]$ necessitates a strategic trade-off between two conflicting objectives: maximizing the aggregate effective rate (Term A) and minimizing the total energy expenditure (Term B). Concurrently, the power splitting coefficient $\mu \left[n\right]$ governs the dynamic prioritization between radar sensing and communication tasks sharing the same spectrum. The primary computational challenge arises from the interference-limited nature of the SINR constraints, where the coupling of power variables in both the numerator and denominator induces strong non-convexity.

This problem remains nonconvex because the rate functions $R_{k,rad}\left[n\right]$ and $R_{k,com}\left[n\right]$ are nonconcave functions of the power variables $P_{u}\left[n\right],\mu \left[n\right]$. We apply the SCA technique by linearizing these rate functions. We use the BCD method to solve it. Therefore, P2.2 is divided into two subproblems as P2.2-1 and P2.2-2.

(38) $$P2.2\text{-}1:\underset{P_{u}\left[n\right]}{\max}2\rho^{\left(j\right)}\sqrt{A}-\rho^{2\left(j\right)}B$$

(38a) $$s.t.\;\left(33p\right)-\left(33r\right)$$

(39) $$P2.2\text{-}2:\underset{\mu \left[n\right]}{\max}2\rho \sqrt{A}-\rho^{2\left(j\right)}B$$

(39a) $$s.t.\;\left(33o\right),\left(33q\right)-\left(33r\right)$$

Mathematically, although the original objective function is non-convex, the logarithmic rate functions contained in Term A are concave with respect to the scalar variable $P_{u}\left[n\right]$. This crucial structural property allows us to apply the Successive Convex Approximation (SCA) method. By replacing the concave rate terms with their first-order Taylor expansions (which serve as rigorous global lower bounds), the problem is transformed into a convex formulation that can be efficiently solved.

Based on this approximation strategy, we first address Subproblem P2.2-1. In this subproblem, the splitting coefficient $\mu \left[n\right]$ is fixed, and we focus on optimizing the transmit power. For the convenience of description, we abbreviate variable symbols, such as. $\mu \left[n\right]\to \mu$. Then, at any given iteration point $P_{u}^{\left(i\right)}$, the $R_{k,rad}\left[n\right]$ and $R_{k,com}\left[n\right]$ can be replaced by their first-order Taylor expansions as follows

(40) $$R_{k,rad}\left[n\right]=\log_{2}\left(1+\frac{\left(1-\mu \right)P_{u}h_{k,rad}}{\mu P_{u}h_{k,rad}+N_{0}B}\right)=\frac{1}{In2}In\left(\frac{P_{u}h_{k,rad}+N_{0}B}{\mu P_{u}h_{k,rad}+N_{0}B}\right)$$

(41) $${\hat{R}}_{k,rad}^{P_{u}}\left[n\right]=\frac{1}{In2}\left(In\left(P_{u}^{\left(i\right)}h_{k,rad}+N_{0}B\right)+\frac{h_{k,rad}\left(P_{u}-P_{u}^{\left(i\right)}\right)}{P_{u}^{\left(i\right)}h_{k,rad}++N_{0}B}-In\left(\mu P_{u}h_{k,rad}+N_{0}B\right)\right)$$

(42) $$R_{k,com}\left[n\right]=\log_{2}\left(1+\frac{\mu P_{u}h_{k,com}}{\left(1-\mu \right)P_{u}h_{k,com}+N_{0}B}\right)=\frac{1}{In2}In\left(\frac{P_{u}h_{k,com}+N_{0}B}{\left(1-\mu \right)P_{u}h_{k,com}+N_{0}B}\right)$$

(43) $${\hat{R}}_{k,com}^{P_{u}}\left[n\right]=\frac{1}{In2}In\left(P_{u}h_{k,com}+N_{0}B\right)-\frac{1}{In2}\left(In\left(\left(1-\mu \right)P_{u}^{\left(i\right)}h_{k,com}+N_{0}B\right)+\frac{\left(1-\mu \right)h_{k,com}\left(P_{u}-P_{u}^{\left(i\right)}\right)}{\left(1-\mu \right)P_{u}^{\left(i\right)}h_{k,com}+N_{0}B}\right)$$

By substituting the exact rate functions with their first-order Taylor expansions derived above, we construct a surrogate global lower bound for the original objective. Consequently, the term A and B are reformulated as the weighted sum of the linearized rate functions $A=\sum_{n=1}^{N}\sum_{k=1}^{K}\omega_{k}\left[n\right]{\hat{R}}_{k,rad}^{P_{u}}\left[n\right]$, while the energy term $B=E_{t}+E_{f}$ remains linear with respect to the transmit power.

The problem P2.2-1 can be reformulated as

(44) $$P2.2\text{-}3:\underset{P_{u}\left[n\right]}{\max}2\rho^{\left(j\right)}\sqrt{A}-\rho^{2\left(j\right)}B$$

(44a) $$s.t.\;0\leq \frac{1}{N}\sum_{n=1}^{N}P_{u}\left[n\right]\leq P_{avg},\forall n$$

(44b) $${\hat{R}}_{k,rad}^{P_{u}}\left[n\right]\leq {\hat{R}}_{k,com}^{P_{u}}\left[n\right],\forall k,n$$

(44c) $$\sum_{n=1}^{N}\sum_{k=1}^{K}\omega_{k}\left[n\right]{\hat{R}}_{k,rad}^{P_{u}}\left[n\right]\leq \sum_{n=1}^{N}\alpha \left[n\right]R_{center}\left[n\right],\forall k,n$$

Problem P2.2-3 is a convex optimization problem and can be solved efficiently using standard convex optimization tools such as CVX.

Following the same algorithmic framework, Subproblem P2.2-2 focuses on optimizing the power splitting coefficient $\mu \left[n\right]$ while holding the total transmit power $P_{u}\left[n\right]$ fixed. Similar to the previous step, the optimization problem is non-convex due to the logarithmic rate functions involved in the constraints and the objective. It is important to note that the rate functions $R_{k,rad}\left[n\right]$ and $R_{k,com}\left[n\right]$ are concave with respect to the splitting coefficient $\mu \left[n\right]$.

For any iteration point $\mu^{\left(i\right)}$, we construct rigorous linear global lower bounds for these rate functions., the $R_{k,rad}\left[n\right]$ and $R_{k,com}\left[n\right]$ can be replaced by their first-order Taylor expansions, which are as follows

(45) $$\begin{array}{l} {\hat{R}}_{k,rad}^{\mu }\left[n\right]=R_{k,rad}^{\left(i\right)}\left[n\right]+{\left.\frac{dR_{k,rad}\left[n\right]}{d\mu }\right|}_{\left(i\right)}\left(\mu -\mu^{\left(i\right)}\right)\\ =\frac{1}{In2}In\left(\frac{P_{u}h_{k,rad}+N_{0}B}{\mu^{\left(i\right)}P_{u}h_{k,rad}+N_{0}B}\right)-\frac{\mu -\mu^{\left(i\right)}}{In2}\frac{P_{u}h_{k,rad}}{\mu P_{u}^{\left(i\right)}h_{k,rad}+N_{0}B} \end{array}$$

(46) $$\begin{array}{l} {\hat{R}}_{k,com}^{\mu }\left[n\right]=R_{k,com}^{\left(i\right)}\left[n\right]+{\left.\frac{dR_{k,com}\left[n\right]}{d\mu }\right|}_{\left(i\right)}\left(\mu -\mu^{\left(i\right)}\right)\\ =\frac{1}{In2}In\left(\frac{P_{u}h_{k,com}+N_{0}B}{\left(1-\mu^{\left(i\right)}\right)P_{u}h_{k,com}+N_{0}B}\right)+\frac{\mu -\mu^{\left(i\right)}}{In2}\frac{P_{u}h_{k,com}}{\left(1-\mu^{\left(i\right)}\right)P_{u}h_{k,com}+N_{0}B} \end{array}$$

The term $A=\sum_{n=1}^{N}\sum_{k=1}^{K}\omega_{k}\left[n\right]{\hat{R}}_{k,rad}^{\mu }\left[n\right]$ and the term $B=E_{t}+E_{f}$. The problem P2.2-2 can be expressed as

(47) $$P2.2\text{-}4:\underset{\mu \left[n\right]}{\max}2\rho^{\left(j\right)}\sqrt{A}-\rho^{2\left(j\right)}B$$

(47a) $$s.t.\;\mu \left[n\right]\in \left[0,1\right],\forall n$$

(47b) $${\hat{R}}_{k,rad}^{\mu }\left[n\right]\leq {\hat{R}}_{k,com}^{\mu }\left[n\right],\forall k,n$$

(47c) $$\sum_{n=1}^{N}\sum_{k=1}^{K}\omega_{k}\left[n\right]{\hat{R}}_{k,rad}^{\mu }\left[n\right]\leq \sum_{n=1}^{N}\alpha \left[n\right]R_{center}\left[n\right],\forall k,n$$

This problem is now linear and can be solved efficiently using CVX. Let γ be the maximum tolerance. The SCA-based Power Optimization Algorithm (Algorithm 1) is described as follows
**Algorithm 1** SCA-based Power Optimization Algorithm1: **Initialize:** $P_{u}^{\left(i\right)}$, $\mu^{\left(i\right)}$, γ, *i* 2: **Repeat** 3: Fix $\mu^{\left(i\right)}$, solve (44) to get solution $P_{u}^{\left(i+1\right)}$; 4. Fix $P_{u}^{\left(i+1\right)}$, solve (47) to get solution $\mu^{\left(i+1\right)}$; 5: Set *i* = *i* + 1; 6: **Until** the growth of objective value is less than γ; 7: **Output:** Power allocation $P=\{P_{u},\;\;\mu \}$

#### 3.2.3. UAV Trajectory Optimization

With fixed task scheduling S and the power allocation P, the UAV’s trajectory optimization subproblem L can be modeled as

(48) $$P2.3:\underset{L}{\max}2\rho^{\left(j\right)}\sqrt{A}-\rho^{2\left(j\right)}B$$

(48a) $$s.t.\;\left(33a\right)-\left(33k\right),\left(33n\right),\left(33q\right)-\left(33r\right)$$

With the scheduling and power allocation variables fixed, the trajectory optimization subproblem remains non-convex. This is because $R_{k,rad}\left[n\right],R_{k,com}\left[n\right],R_{center}\left[n\right]$ are nonconvex functions of the UAV’s trajectory with respect to $q\left[n\right]$, $H_{u}\left[n\right]$, while the propulsion energy term in the objective is a non-convex function of $v\left[n\right],a\left[n\right]$.

To make problem P2.3 tractable, we adopt a two-stage strategy. First, we introduce auxiliary slack variables to decouple the trajectory variables from the non-convex fractional terms. Second, we apply the SCA framework. Specifically, since the squared Euclidean distance is a convex function with respect to the UAV location, its first-order Taylor expansion serves as a rigorous global lower bound.

From (19)–(26), we obtain

(49) $$R_{k,rad}\left[n\right]=\log_{2}\left(1+\frac{P_{rad}\left[n\right]\beta_{r}}{P_{com}\left[n\right]\beta_{r}+N_{0}Bd_{k}^{4}\left[n\right]}\right)=\frac{1}{In2}In\left(1+\frac{\left(1-\mu \right)P_{u}\beta_{r}}{\mu P_{u}\beta_{r}+N_{0}Bd_{k}^{4}\left[n\right]}\right)$$

(50) $$R_{k,com}\left[n\right]=\log_{2}\left(1+\frac{\mu P_{u}h_{k,com}}{\left(1-\mu \right)P_{u}h_{k,com}+N_{0}B}\right)=\frac{1}{In2}In\left(1+\frac{\mu P_{u}\beta_{0}}{\left(1-\mu \right)P_{u}\beta_{0}+N_{0}Bd_{k}^{2}}\right)$$

(51) $$R_{center}\left[n\right]=\log_{2}\left(1+\frac{P_{u}\left[n\right]\beta_{0}}{N_{0}Bd_{c}^{2}\left[n\right]}\right)=\frac{1}{In2}In\left(1+\frac{P_{u}\left[n\right]\beta_{0}}{N_{0}Bd_{c}^{2}\left[n\right]}\right)$$

Furthermore, we observe that the original rate functions in (49)–(51) are logarithmic functions of the distance. To simplify the expression and aid in the convexification process, we introduce a lemma.

**Lemma 1.**  *Let* a>0*,* b>0 *and* c>0 *, then the function*

(52) $$f\left(Z\right)=In\left(1+\frac{a}{b+cZ}\right)$$

*at a local point* $Z^{\left(j\right)}$ *, its lower bound can be written as:*

(53) $$In\left(1+\frac{a}{b+cZ}\right)\geq In\left(1+\frac{a}{b+cZ^{\left(j\right)}}\right)-\frac{ac}{\left(b+cZ^{\left(j\right)}\right)\left(a+b+cZ^{\left(j\right)}\right)}\left(Z-Z^{\left(j\right)}\right)$$

**Proof.**  Please refer to Appendix A. □

Following this result, we apply Lemma 1 to linearize the nonconvex components in Equations (49)–(51), which exhibit a similar structure to (52). In the following, we use the shorthand notation d for $d\left[n\right]$ to simplify the expressions. Specifically, (49) and (50) are lower-bounded by their first-order Taylor approximations constructed at the point $d_{k}^{2\left(i\right)}={\left\|q^{\left(i\right)}-g_{k}\right\|}^{2}+{\left(H_{u}^{\left(i\right)}-H_{k}\right)}^{2}$ and $d_{c}^{2\left(i\right)}={\left\|q^{\left(i\right)}-g_{c}\right\|}^{2}+{\left(H_{u}^{\left(i\right)}-H_{c}\right)}^{2}$. The function in (50) and (51) is reformulated as

(54) $$R_{k,com}\left[n\right]\geq {\tilde{R}}_{k,com}\left[n\right]=R_{k,com}^{\left(i\right)}\left[n\right]+F_{k,com}^{\left(i\right)}\left(d_{k}^{2}-d_{k}^{2\left(i\right)}\right)$$

(55) $$R_{center}\left[n\right]\geq {\tilde{R}}_{center}\left[n\right]=R_{center}^{\left(i\right)}\left[n\right]+F_{center}^{\left(i\right)}\left(d_{c}^{2}-d_{c}^{2\left(i\right)}\right)$$

where

(56) $$F_{k,com}^{\left(i\right)}=\frac{1}{In2}\frac{-N_{0}B\cdot \mu \cdot P_{u}\beta_{0}}{\left(P_{u}\beta_{0}+N_{0}Bd_{k}^{2\left(i\right)}\right)\left(\left(1-\mu \right)\cdot P_{u}\beta_{0}+N_{0}Bd_{k}^{2\left(i\right)}\right)}$$

(57) $$F_{center}^{\left(i\right)}=\frac{1}{In2}\frac{-P_{u}\beta_{0}}{\left(P_{u}\beta_{0}+N_{0}Bd_{c}^{2}\right)d_{c}^{2}}$$

(58) $$R_{k,com}^{\left(i\right)}=\log_{2}\left(1+\frac{P_{com}\left[n\right]\beta_{r}}{P_{rad}\left[n\right]\beta_{r}+N_{0}Bd_{k}^{2(i)}\left[n\right]}\right)$$

(59) $$R_{center}^{\left(i\right)}\left[n\right]=\log_{2}\left(1+\frac{P_{u}\left[n\right]\beta_{0}}{N_{0}Bd_{c}^{2(i)}\left[n\right]}\right)$$

Then, we apply a linear form to the function (49):

(60) $${\tilde{R}}_{k,rad}\left[n\right]\approx R_{k,rad}^{\left(i\right)}\left[n\right]+F_{k,rad}^{\left(i\right)}\left(d_{k}^{2}-d_{k}^{2\left(i\right)}\right)$$

where

(61) $$F_{k,rad}^{\left(i\right)}=\frac{1}{In2}\frac{-2N_{0}Bd_{k}^{2}P_{u}\beta_{r}\left(1-\mu \right)}{\left(N_{0}Bd_{k}^{4}+P_{u}\beta_{r}\right)\left(\mu \cdot P_{u}\beta_{r}+N_{0}Bd_{k}^{4}\right)}$$

Define $D_{c}^{\left(i\right)}=d_{c}^{2}-d_{c}^{2\left(i\right)}$ and $D_{k}^{\left(i\right)}=d_{k}^{2}-d_{k}^{2\left(i\right)}$. Because these terms still contain quadratic components, they are not directly compatible with the CVX modeling rules. To handle this issue, we introduce two auxiliary variables $\tau_{c}$ and $\tau_{k}$ to relax the functions (54), (55), (60), which can be written as

(62) $${\tilde{R}}_{k,com}\left[n\right]=R_{k,com}^{\left(i\right)}\left[n\right]+F_{k,com}^{\left(i\right)}\tau_{c}$$

(63) $${\tilde{R}}_{center}\left[n\right]=R_{center}^{\left(i\right)}\left[n\right]+F_{center}^{\left(i\right)}\tau_{c}$$

(64) $${\tilde{R}}_{k,rad}\left[n\right]\approx R_{k,rad}^{\left(i\right)}\left[n\right]+F_{k,rad}^{\left(i\right)}\tau_{k}$$

where

(65) $$\tau_{k}\geq D_{k}^{\left(i\right)}=d_{k}^{2}-d_{k}^{2\left(i\right)}$$

(66) $$\tau_{c}\geq D_{c}^{\left(i\right)}=d_{c}^{2}-d_{c}^{2\left(i\right)}$$

Due to the non-convexity of constraint (33o), we employ the first-order Taylor expansion to derive a linear lower bound for the coverage constraint at the local point $H_{u}^{\left(i\right)}\left[n\right]$. The resulting convexified constraint is expressed as:

(67) $${\left(H_{u}\left[n\right]\tan\theta \right)}^{2}\geq {\tilde{H}}_{u}\left[n\right]={\left(H_{u}^{\left(i\right)}\left[n\right]\tan\theta \right)}^{2}+2\tan^{2}\theta H_{u}^{\left(i\right)}\left[n\right]\left(H_{u}\left[n\right]-H_{u}^{\left(i\right)}\left[n\right]\right)$$

For the non-convex item $E_{f}$, we introduce a slack variable $\tau_{e}^{2}\left[n\right]\leq {\left\|v\left[n\right]\right\|}^{2}$. Then $E_{f}$ can be rewritten as

(68) $${\tilde{E}}_{f}=\sum_{n=1}^{N}\delta \left(c_{1}{\left\|v\left[n\right]\right\|}^{3}+\frac{c_{2}}{\left\|\tau_{e}\left[n\right]\right\|}\left(1+\frac{{\left\|a\left[n\right]\right\|}^{2}}{g^{2}}\right)\right)$$

For any fixed point $v^{\left(i\right)}\left[n\right]$, ${\left\|v\left[n\right]\right\|}^{2}$ can be rewritten as

(69) $${\left\|v\left[n\right]\right\|}^{2}\geq v^{lb}\left[n\right]={\left\|v^{\left(i\right)}\left[n\right]\right\|}^{2}+2v^{\left(i\right)}{\left[n\right]}^{T}\left(v\left[n\right]-v^{\left(i\right)}\left[n\right]\right)$$

Based on (59)–(61), (65) and (66), the term $A=\sum_{n=1}^{N}\sum_{k=1}^{K}\omega_{k}\left[n\right]{\tilde{R}}_{k,rad}\left[n\right]$ and the term $B=E_{t}+{\tilde{E}}_{f}$, the problem P2.3 can be equivalently expressed as the following:

(70) $$P2.4:\underset{L}{\max}2\rho^{\left(j\right)}\sqrt{A}-\rho^{2\left(j\right)}B$$

(70a) $$s.t.\;\left(33a\right)-\left(33k\right)$$

(70b) $$R_{k,rad}\left[n\right]\leq R_{k,com}\left[n\right],\forall k,n$$

(70c) $$\sum_{n=1}^{N}\sum_{k=1}^{K}\omega_{k}\left[n\right]{\tilde{R}}_{k,rad}\left[n\right]\leq \sum_{n=1}^{N}\alpha \left[n\right]{\tilde{R}}_{center}\left[n\right],\forall k,n$$

(70d) $$\tau_{e}^{2}\left[n\right]\leq {\left\|v\left[n\right]\right\|}^{2},\tau_{e}\left[n\right]\geq V_{\min},\forall k,n$$

(70e) $$\omega_{k}\left[n\right]{\left\|q\left[n\right]-g_{k}\right\|}^{2}\leq {\tilde{H}}_{u}\left[n\right],\forall k,n$$

which is a convex problem and can be solved through CVX.

### 3.3. AO-Based Two-Layer-Three-Stages Optimization

To solve the original problem P1, we propose a two-layer three-stage iterative optimization algorithm that alternately optimizes the three subproblems P2.1, P2.2 and P2.3 until the objective value converges, let ε represent the maximum tolerance, as shown in Algorithm 2.
**Algorithm 2** AO-Based Two-layer-Three-Stages Optimization1: **Initialize:** Convergence tolerance ε, iteration index *j* = 0, initial energy efficiency $\rho^{\left(0\right)}=0$. 2: **Initialize:** Feasible task scheduling **S**, power allocation **P**, and trajectory **L.**
3: **repeat**                                                                                                                   ▷ *Outer Loop* 4. Set inner iteration index *i* = 0 5: Initialize inner variables: $\left\{S^{\left(0\right)},\;P^{\left(0\right)},\;L^{\left(0\right)}\right\}⟵\{S,P,L\}$ 6: **while** not converged **do**                                                             ▷ *Inner Loop: BCD Method* 7: *i* ⟵ *i* + 1 8: Fix $P^{\left(i-1\right)},\;L^{\left(i-1\right)}$ and $\rho^{\left(j\right)}$, solve (**P2.1**) to obtain $S^{\left(i\right)}$ 9: Fix $S^{i}$, $L^{\left(i-1\right)}$ and $\rho^{\left(j\right)}$, solve (**P2.2**) to obtain $P^{\left(i\right)}$ 10: Fix $S^{i}$, $P^{\left(i\right)}$ and $\rho^{\left(j\right)}$, solve (**P2.3**) to obtain $L^{\left(i\right)}$ 11: **end while** 12: Update variables: $\left\{S,P,L\right\}⟵\left\{S^{\left(i\right)},\;P^{\left(i\right)},\;L^{\left(i\right)}\right\}$ 13: **Update energy efficiency:**
$$\;\eta_{SEE}=\;\frac{\sum_{n=1}^{N}\sum_{k=1}^{K}\omega_{k}\left[n\right]R_{k,rad}[n]}{E_{t}+E_{f}}$$14: *j* ⟵ *j* + 1 15: **until** $\left|\rho^{\left(j\right)}-\;\rho^{\left(j-1\right)}\right|\;\leq \varepsilon$ 16: **Output:** Optimal solution $S^{*},\;P^{*},\;L^{*}$ and max EE. 

## 4. Simulation Results

In this section, we conduct simulation experiments to verify the logical soundness of the proposed system model and to evaluate the effectiveness of the optimization scheme. The parameter settings used in these simulations are summarized in Table 1.

We assume a square region of 1200 m×1200 m where 12 sensing target nodes are randomly distributed, and the data collection center is located at the center of the area. To get the optimal UAV trajectory, we define the initial UAV flight trajectory as a circular route centered around the data collection center with a radius of r. Additionally, the initial trajectory and velocity of the UAV can be expressed as follows

(71) $$r=\frac{\max\left\|g_{c}-g_{k}\right\|+\min\left\|g_{c}-g_{k}\right\|}{2}$$

(72) $$v_{init}\left[n\right]=\left(\frac{2\pi r}{T}\cos\psi ,\frac{2\pi r}{T}\sin\psi ,0\right),\forall n$$

(73) $$\psi =\frac{2\pi \left(n-1\right)}{N-1},\forall n$$

(74) $$q_{init}\left[n\right]=\left(r\cos\psi ,r\sin\psi ,H_{init}\right),\forall n$$

(75) $$H_{init}=\frac{H_{u}^{\min}+H_{u}^{\max}}{2}$$

**Figure 2:**
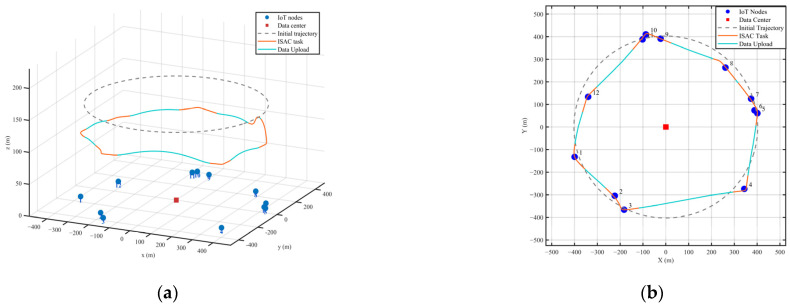
(**a**) Optimized 3D trajectory and task scheduling; (**b**) Top view of optimized 3D trajectory and task scheduling.

Under the given initial flight configuration, the UAV’s transmit power is initialized as $P_{u}\left[n\right]=1W$, and the power allocation factor is set as $\mu \left[n\right]=0.5$. Based on these settings, Algorithm 2 outputs an optimized three-dimensional trajectory, as illustrated in Figure 2. The numerical labels adjacent to the nodes indicate their respective indices. In Figure 2a, the orange and blue segments depict the UAV’s flight path during the ISAC task and the data upload task, respectively. The optimized trajectory exhibits multiple altitude adjustments, which, combined with the coverage constraint (15), suggest an adaptive strategy to balance sensing coverage and link quality. Moreover, under the fixed radar sensing angle of θ=30°, a higher flight altitude means a broader ground detection range. It is worth noting that despite these altitude adjustments, the UAV’s altitude remains within a relatively stable range without drastic fluctuations. This reflects an energy-aware design, as frequent or large changes in altitude would increase propulsion energy consumption, thereby reducing overall energy efficiency.

The top view of the 3D trajectory and task scheduling is illustrated in Figure 2a. It is evident that the UAV’s flight path is governed by the principle of balancing sensing demands with energy expenditure. Specifically, the UAV maintains close proximity to the nodes during ISAC to achieve an optimal trade-off between perceptual gains and communication power costs. This behavior, combined with direct high-speed transit between nodes, forms a comprehensive strategy to minimize overall energy consumption while maintaining mission effectiveness.

**Figure 3:**
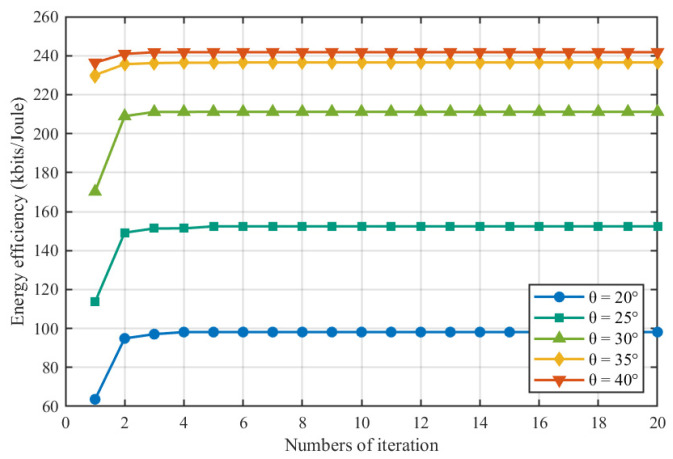
Energy efficiency Comparison at different detection angles.

Figure 3 illustrates the convergence behavior of the proposed Algorithm 2 under different sensing beam angles θ. It can be observed that, for all considered angles, the algorithm converges to a stable energy efficiency value within approximately 3–5 outer iterations. Taking θ=20° as the baseline, the converged energy efficiency is about 98 kbits/joule. When the sensing angle increases to θ=25°, the final energy efficiency improves to 154.2 kbits/joule, 55.3% higher than the baseline. For θ=30°, the energy efficiency is further increased to 211.2 kbits/joule, corresponding to an improvement of 115.1% over θ=20°. When the sensing angle is enlarged to θ=35° and θ=40°, the achieved energy efficiencies reach 236.5 kbits/joule and 241.8 kbits/joule, respectively, which represent gains of 141.0% and 146.3% compared with the baseline. These results indicate that appropriately increasing the sensing angle can significantly enlarge the set of simultaneously observable IoT nodes and thus enhance the number of effective sensing bits obtained per unit energy. However, as the sensing angle continues to increase, the additional gain in energy efficiency tends to saturate, since most IoT nodes have already been covered.

**Figure 4:**
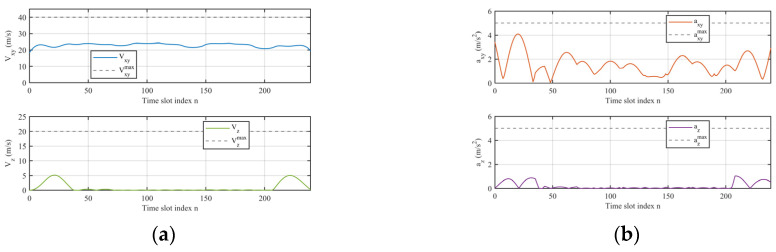
(**a**) Optimized speeds of the UAV; (**b**) Optimized acceleration of the UAV.

Figure 4a illustrates the horizontal and vertical velocity profiles of the UAV over the flight period. The horizontal velocity $V_{xy}$ remains within a relatively narrow range around its energy-efficient cruising value and is always well below the maximum speed constraint $V_{xy}^{\max}=40\;m/s$. Only small fluctuations are observed along the flight, which indicates that the optimized trajectory tends to keep a nearly constant horizontal speed while the UAV circulates above the IoT nodes. This behavior is consistent with the adopted propulsion energy model (30), where the flying power consumption is a convex function of the velocity, so that staying close to the optimal cruising speed is beneficial for maximizing the overall energy efficiency.

The vertical velocity $V_{z}$ is equal to zero for most time slots and exhibits noticeable non–zero values only in a few short intervals at the beginning and near the end of the flight period. This means that the UAV flies at an almost constant altitude during the data collection phase, and only performs ascending/descending maneuvers when it needs to adjust its altitude to satisfy the coverage constraint or to return to the initial height. Moreover, the peaks of $V_{z}^{\max}=20\;m/s$, which confirms that the vertical motion strictly respects the imposed kinematic limits.

Figure 4b further shows the corresponding horizontal and vertical acceleration components, $a_{xy}$ and $a_{z}$. Compared with the velocity curves, the horizontal acceleration exhibits larger oscillations in several local segments, but its magnitude always remains under the maximum allowed value $a_{xy}^{\max}=5{\;m/s}^{2}$. These horizontal accelerations primarily occur during segments with significant trajectory direction changes, such as when the UAV moves between nodes 1 and 3, where notable variations in altitude coincide with pronounced acceleration fluctuations. During other intervals, the acceleration remains close to zero. This pattern demonstrates that the proposed algorithm optimization method strategically increases acceleration only when necessary to enhance ISAC performance, while avoiding unnecessary consumption, thereby conserving propulsion energy.

The vertical acceleration is close to zero for the majority of the period and only shows several small peaks around the intervals where non–zero vertical velocities appear. This is consistent with the previous observation that the UAV mainly flies at a constant altitude and only adjusts its height in a few short phases.

**Figure 5:**
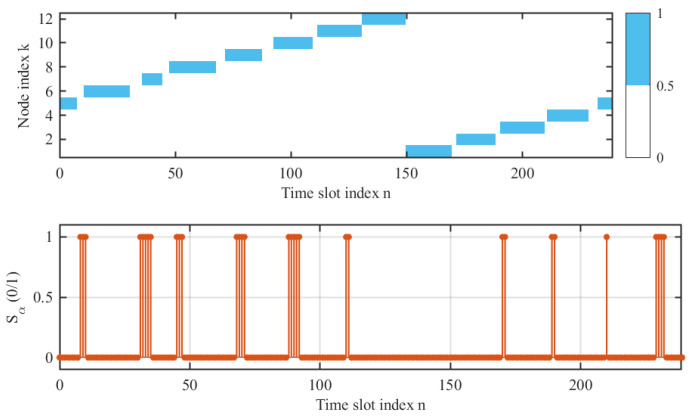
Task scheduling of the UAV.

Figure 5 illustrates the task scheduling of the UAV across time slots. The upper subplot depicts the slots allocated for executing ISAC tasks towards the ground nodes, while the lower subplot corresponds to the slots dedicated to data transmission. It can be observed that the data upload tasks are strategically scheduled during the transit phases between different nodes. This scheduling strategy ensures that all available time slots are seamlessly occupied, leaving no idle resources and thereby enhancing overall system efficiency.

**Figure 6:**
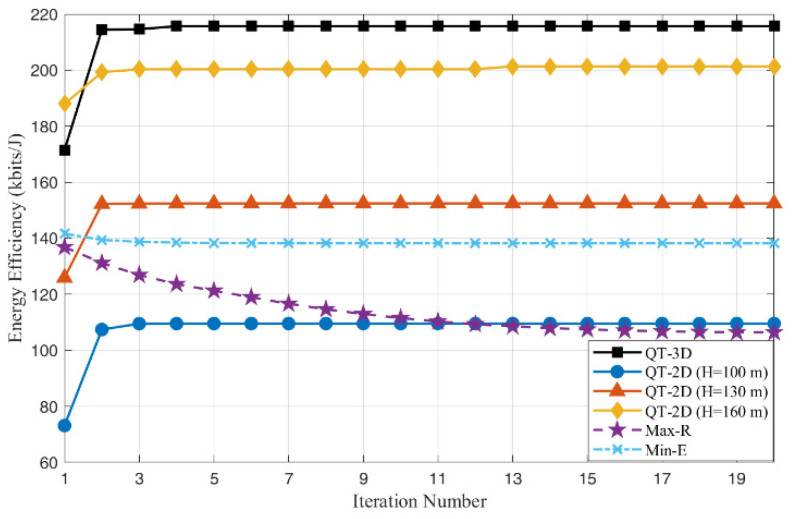
Different Energy efficiency algorithm comparison.

The Min-E algorithm evaluates energy efficiency under the flight trajectory designed to minimize total energy consumption. In contrast, the Max-R algorithm assesses energy efficiency under the trajectory that achieves the maximum sensing data volume. QT-3D represents our proposed Algorithm 2, which jointly optimizes the 3D trajectory, transmit power, and task scheduling. QT-2D serves as a benchmark variant of Algorithm 2, where the UAV is constrained to a fixed flight altitude. Figure 6 illustrates the convergence behaviors of these algorithms. As shown in Figure 6, the proposed QT-3D algorithm demonstrates superior performance compared to the benchmark schemes. This algorithm achieves the highest energy efficiency, converging to approximately 215.2 kbits/joule. By exploiting the degrees of freedom in the vertical dimension, the UAV dynamically adjusts its altitude to balance path loss reduction with improved LoS probability and coverage, thereby maximizing the system’s total energy efficiency. QT-2D underperforms QT-3D by approximately 7.1%, confirming that fixing the altitude limits the UAV’s ability to fully exploit spatial resources for performance gain. The Max-R algorithm exhibits a distinct downward trend in energy efficiency. This decline occurs because the algorithm aggressively pursues a higher sensing rate by using maximum transmit power and high-speed maneuvers. Consequently, the energy consumption increases disproportionately faster than the data rate, leading to a continuous reduction in overall efficiency. This result highlights that solely maximizing throughput is inefficient for green communications. The Min-E algorithm successfully reduces energy costs, it does not optimize for data throughput, resulting in a lower energy efficiency.

**Figure 7:**
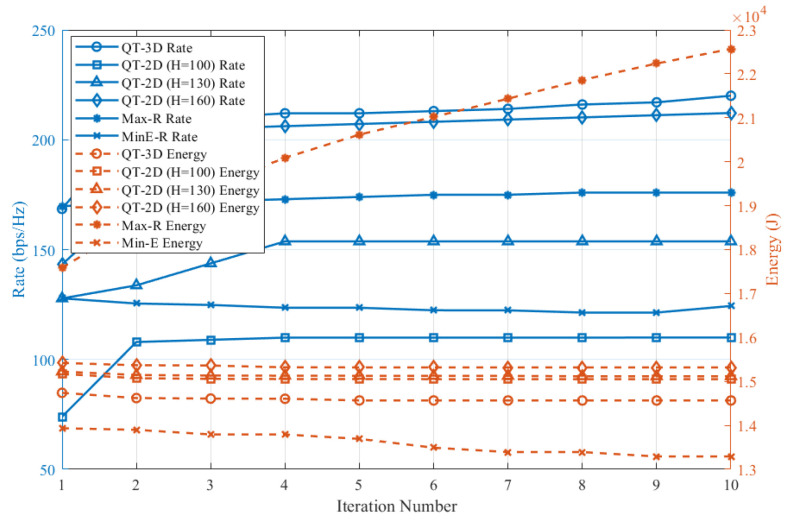
The term A and B Convergence Comparison.

Figure 7 illustrates the convergence behavior of the sensing rate (left y-axis) and the total energy consumption (right y-axis) over 10 iterations for all considered algorithms. We examine the convergence of the sensing rate and energy consumption separately. As can be observed, the proposed QT-3D achieves a steady increase in sensing rate from 168 bit/Hz to 207 bit/Hz, while its energy consumption rises moderately from 148 kJ to approximately 145 kJ, demonstrating a well-balanced trade-off between sensing performance and energy expenditure.

## 5. Conclusions

This paper has presented an energy-aware design framework for UAV-assisted integrated sensing and communication systems. The core contribution lies in the introduction of sensing energy efficiency (SEE) as a pivotal system-level metric, which effectively unifies the conflicting objectives of high sensing performance and sustainable operation under a stringent onboard energy budget. To maximize the SEE, we developed a holistic optimization formulation that jointly designs the UAV’s 3D trajectory, task scheduling, and power allocation, subject to practical kinematic and coverage constraints. A novel and efficient two-layer algorithm based on the Dinkelbach method, block coordinate descent, and successive convex approximation was proposed to solve this challenging nonconvex problem. Simulation results validate the superiority of the framework, demonstrating that the optimized solution intelligently balances sensing coverage and energy expenditure, leading to significant performance gains over conventional schemes. While the solution is obtained via iterative convex approximation, which yields a local optimum, the consistent and substantial improvements confirm the practical effectiveness and advancement of the proposed design. This work establishes a solid foundation for energy-efficient UAV-ISAC systems, with future extensions potentially encompassing mobile targets, multi-UAV cooperation, and dynamic channel environments. Future extensions will focus on validating the framework under communication environmental uncertainties (e.g., wind, GPS errors) and integrating AI-based techniques for dynamic adaptation, alongside exploring mobile targets, multi-UAV cooperation, and dynamic channel environments. These explorations will further advance the convergence of communication, sensing, computing, control, and intelligence in next-generation wireless networks.

## Figures and Tables

**Table 1:** Simulation Parameters.

Parameter	Value
number of sensing nodes	K=12
total flight time of the UAV	T=120 s
time interval	δ=0.5 s
maximum horizontal speed of the UAV	$V_{xy}^{\max}=40\;m/s$
minimum horizontal speed of the UAV	$V_{xy}^{\min}=0\;m/s$
maximum vertical speed of the UAV	$V_{z}^{\max}=20\;m/s$
maximum horizontal acceleration of the UAV	$a_{xy}^{\max}=5{\;m/s}^{2}$
maximum vertical acceleration of the UAV	$a_{z}^{\max}=5{\;m/s}^{2}$
minimum flight altitude of the UAV	$H_{\min}=100\;m$
maximum flight altitude of the UAV	$H_{\max}=200\;m$
maximum detection angle of the UAV	θ=30°
average communication power of the UAV	$P_{avg}=10\;W$
propulsion parameters	$c_{1}=9.26\times 10^{-4}$
propulsion parameters	$c_{2}=2250$
carrier frequency	$f_{c}=3.5\;GHz$
bandwidth	B=30 MHz
noise power spectral density	$N_{0}=-160\;dBmW/Hz$
RCS of the sensing target	$\sigma =10{\;m}^{2}$
antenna gain of the UAV transmitter	$G_{t}=17\;dBi$
antenna gain of the UAV receiver	$G_{r}=17\;dBi$
communication receiver antenna gain	$G_{c}=0\;dBi$
maximum error tolerance	$\varepsilon =10^{-3},\gamma =10^{-3}$

## Data Availability

The original contributions presented in this study are included in the article. Further inquiries can be directed to the corresponding author.

## Appendix A. Proof of Lemma 1

**Lemma 1.** *Let* a>0*,* b>0 *and* c>0 *, then the function*

(A1) $$f\left(Z\right)=In\left(1+\frac{a}{b+cZ}\right)$$

*at a local point* $Z^{\left(j\right)}$ *, its lower bound can be written as:*

(A2) $$In\left(1+\frac{a}{b+cZ}\right)\geq In\left(1+\frac{a}{b+cZ^{\left(j\right)}}\right)-\frac{ac}{\left(b+cZ^{\left(j\right)}\right)\left(a+b+cZ^{\left(j\right)}\right)}\left(Z-Z^{\left(j\right)}\right)$$

**Proof.** We can get

(A3) $$f\left(Z\right)=In\left(1+\frac{a}{b+cZ}\right)=In\left(\frac{b+cZ+a}{b+cZ}\right)=In\left(b+cZ+a\right)-In\left(b+cZ\right)$$

Then, the first-order and second-order derivatives of $f\left(Z\right)$ can be written as

(A4) $$f\left(Z\right)=In\left(b+cZ+a\right)-In\left(b+cZ\right)$$

(A5) $$f^{\prime }\left(Z\right)=\frac{1}{X+a}-\frac{1}{X}=-\frac{ac}{\left(b+cZ\right)\left(b+cZ+a\right)}$$

(A6) $$f^{\prime \prime }\left(Z\right)=\frac{ac^{2}\left(2cZ+a+2b\right)}{{\left(b+cZ\right)}^{2}{\left(b+cZ+a\right)}^{2}}$$

Since a>0, b>0, c>0 and Z>0, we can get $f^{\prime \prime }\left(Z\right)>0$. Therefore, we can use the linear expression obtained by its first-order Taylor expansion as the lower bound of $f\left(Z\right)$ at the local point $Z^{\left(j\right)}$ which can be written as

(A7) $$f\left(Z\right)\geq f\left(Z^{\left(j\right)}\right)+f^{\prime }\left(Z^{\left(j\right)}\right)\left(Z-Z^{\left(j\right)}\right)$$

 □

## Appendix B. Proof of Equivalence for the Quadratic Transform

Let the original fractional objective function be denoted as $f\left(Χ\right)=\frac{A\left(Χ\right)}{B\left(Χ\right)}$,where $A\left(Χ\right)$ is the non-negative numerator (Total Rate) and $B\left(Χ\right)$ is the positive denominator (Total Energy). The proposed auxiliary function is defined as:

(A8) $$F\left(Χ,\rho \right)=2\rho \sqrt{A\left(Χ\right)}-\rho^{2}B\left(Χ\right)$$

We first treat Χ as fixed and optimize F with respect to ρ. Since F is a quadratic concave function of ρ, we set its partial derivative to zero to find the optimal $\rho^{*}$:

(A9) $$\frac{\partial F\left(Χ,\rho \right)}{\partial \rho }=2\sqrt{A\left(Χ\right)}-2\rho B\left(Χ\right)=0$$

Solving for ρ, we obtain the optimal closed-form solution:

(A10) $$\rho^{*}=\frac{\sqrt{A\left(Χ\right)}}{B\left(Χ\right)}$$

Substituting $\rho^{*}$ back into the auxiliary function F, we get:

(A11) $$\begin{array}{c} F\left(Χ,\rho \right)=2\frac{\sqrt{A\left(Χ\right)}}{B\left(Χ\right)}\sqrt{A\left(Χ\right)}-{\left(\frac{\sqrt{A\left(Χ\right)}}{B\left(Χ\right)}\right)}^{2}B\left(Χ\right)\\ =\frac{2A\left(Χ\right)}{B\left(Χ\right)}-\frac{A\left(Χ\right)}{B\left(Χ\right)}\;\;\;\;\;\;\;\;\;\;\;\;\;\;\;\;\;\;\;\\ =\frac{A\left(Χ\right)}{B\left(Χ\right)}=f\left(Χ\right)\;\;\;\;\;\;\;\;\;\;\;\;\;\;\;\;\;\;\;\;\;\;\; \end{array}$$

Consequently, the parametric problem (P2) is equivalent to the original fractional problem (P1). This completes the proof.
